# Enhanced release of IgE-dependent early phase mediators from nasal polyp tissue

**DOI:** 10.1186/1476-9255-6-11

**Published:** 2009-04-20

**Authors:** Joke Patou, Gabriele Holtappels, Karen Affleck, Philippe Gevaert, Claudina Perez-Novo, Paul Van Cauwenberge, Claus Bachert

**Affiliations:** 1 Upper Airways Research Laboratory, Department of Otorhinolaryngology, Ghent University, Ghent, Belgium; 2 GSK, Stevenage, SG1 2NY, UK

## Abstract

**Background:**

The mast cell is a crucial effector cell in allergic rhinitis and other inflammatory diseases. During the acute allergic reaction preformed mediators such as histamine, but also *de novo *produced mediators such as leukotrienes (LTC_4_/D_4_/E_4_) and prostaglandins (PGD_2_) are released. Mast cells represent targets for therapeutic intervention, and thus a human ex-vivo model to stimulate mast cells taken from mucosal sites would be instrumental for drug intervention studies. We have aimed to activate mast cells within ex-vivo human nasal tissue by IgE/anti-IgE specific (ε chain specific) stimulations and in this respect to test the usability of nasal polyps versus inferior turbinates

**Methods:**

Biopsy samples were collected from patients with nasal polyps and inferior turbinates from patients who underwent sinus or septal surgery. Tissue fragments were primed with IgE 1 μg/ml for 60 minutes and then stimulated for 30 minutes with tissue culture medium (negative control), anti-IgE 10 μg/ml, anti-IgE 30 μg/ml and ionomycin 10 μM (positive control). Histamine, leukotrienes and PGD_2 _were measured in supernatants. To help provide an understanding of the extent of the response, the number of tryptase and FcεRIα positive cells was evaluated by means of immunohistochemistry and the FcεRIα-chain was measured by means of quantitative PCR in the nasal polyp and inferior turbinate tissues. Finally, the correlation between IgE concentrations in the nasal tissue and the release of mediators was analysed.

**Results:**

Stimulations with anti-IgE on IgE-primed nasal tissue fragments lead to a concentration-dependent release of histamine, leukotrienes and PGD_2_. The release of these early phase mediators was significantly higher in nasal polyps compared to inferior turbinates, although tryptase, FcεRIα positive cells and FcεRIα-chain transcripts were equally present in both groups. No correlation was found between baseline concentrations of IgE, and the release of histamine, LTC_4_/LTD_4_/LTE_4 _and PGD_2 _after stimulation.

**Conclusion:**

This human nasal challenge model mimics the allergic early phase reaction. The release of histamine, cys-leukotrienes and PGD_2 _was significantly higher in nasal polyps versus inferior turbinates, however, this observation could not be explained by differences in mast cell or FcεRI+ cell numbers.

## Background

Mast cells play a crucial role in allergic rhinitis and other inflammatory responses. Positioned at mucosal surfaces, these cells are situated to be among the first to encounter antigens that elicit allergic reactions. Interaction of multivalent allergens with cell-bound specific immunoglobuline E (IgE) leads to cross-linking of the high affinity IgE receptor (FcεRI), which is primarily expressed on mast cells and basophils. First, this results in the immediate release of the content of mast cell secretory granules, which includes preformed mediators such as histamine, neutral proteases and proteoglycans and second, it results in the *de novo *synthesis of mediators including the products of the arachidonic acid metabolism, such as prostaglandin D_2 _(PGD_2_) and sulfidopeptidyl leukotrienes C_4_/D_4_/E_4_, and the production of several cytokines (i.e. IL-4, IL-5, IL-6, TNF-α, IL-13) [1,2]. During the acute allergic reaction mainly preformed mediators such as histamine, but also newly produced mediators such as leukotrienes (LTC_4_/D_4_/E_4_) and PGD_2 _are released [3]. These mediators initiate rapid vascular permeability, leading to plasma extravasation and tissue edema, mucous overproduction and leukocyte recruitment.

Most early studies of mast cells rely on the use of transformed mast cells from murine mastocytoma cells [4,5]. Currently, it is possible to grow human mast cells *in vitro*. Interleukin (IL)-3, IL-6 and stem cell factor (SCF) may act on hematopoietic stem cells present in bone marrow, umbilical cord blood, fetal liver or peripheral blood and make it possible to grow large numbers of committed mast cell precursors. These cells express high levels of c-kit receptor and FcεRI [6]. Furthermore, several mast cell lines such as HMC-1 [7] or LAD-1/2 [8] are available to study mast cell biology. The use of murine cells, the addition of several factors to grow human mast cells, or the use of human mast cell lines may induce responses different from primary *in vivo *tissue mast cells.

Considerable difficulties exist to isolate and stimulate mast cells from nasal tissue; especially the limited amount of tissue extracted after surgery (turbinotomy) and the low number of mast cells isolated from nasal tissue, may give problems to stimulate nasal mast cells directly [9]. To study nasal mast cells, stimulations have been done in enzymatic dispersed nasal polyp tissue [10,11]. Accessibility of nasal polyp tissue allows for easy assessment of interaction between different cell types in an inflammatory environment; however, enzymatic digestion of tissue may possible damage receptors and the comparability of results obtained from nasal polyp stimulations to inferior turbinate stimulations is not clear.

We therefore aimed to study mast cells and basophils in their tissue environment by using IgE/anti-IgE driven (ε chain specific) stimulations in human nasal tissue explants without enzymatic digestion to closely mimic the *in vivo *situation. Second we wanted to test the usability of nasal polyps versus inferior turbinates in this respect, as polyp tissue is easier to obtain in larger quantities. Finally, we aimed to explain differences in the response between tissues, and studied tryptase and FcεRIα + cell numbers, as well as baseline concentrations of IgE in relation to mast cell responses [12].

## Methods

### Patients

Nasal tissue was obtained from 8 polyp patients and 8 control patients at the Department of Otorhinolaryngology of the University Hospital of Ghent. The ethical committee of the Ghent University Hospital approved the study and all patients gave their written informed consent prior to inclusion in the study.

None of the subjects received intranasal corticosteroids, anti-histamines or anti-leukotrienes, oral and intranasal decongestants or intranasal anticholinergics within 1 week prior to surgery and none of the subjects received oral and/or intramuscular corticosteroids within 4 weeks prior to surgery. For female subjects pregnancy or lactation was excluded.

The control group was composed of samples collected from the inferior turbinates from patients undergoing septal surgery and/or turbinotomy because of nasal obstruction, unrelated to this study.

Nasal polyp samples were collected during functional endoscopic sinus surgery. Nasal polyposis was diagnosed based on symptoms, clinical examination, nasal endoscopy, and sinus computed tomography (CT) scan according to the EP^3^OS guidelines [13].

The atopic status of all patients was evaluated by skin prick tests with a standard panel of 14 inhalant allergens, including negative (NaCl solution) and positive controls (10 mg/ml histamine solution). The reaction to a skin prick test was considered positive if the wheal area caused by the allergen was greater than 7 mm^2 ^(diameter >3 mm). Patient characteristics are displayed in table 1.

**Table 1:** Patient characteristics

	**Inferior turbinates**	**Nasal polyps**
N	8	8

Age (median, range)	36.5 (17–47)	38.5 (18–54)

Female/male	2/6	4/4

Asthma in history	1/8	0/8

Skin prick test-positive	0/8	2/8

Aspirin intolerance	0/8	0/8

Smoking	1/8	1/8

The nasal tissue collected during surgery was immediately transported to the laboratory, partly snap frozen in liquid nitrogen, and stored at -80°C until analysis for immunohistochemistry, IgE measurement and PCR. The remaining tissue was used for the *ex-vivo *stimulations.

### Mechanical disruption and stimulations of human nasal tissue

The human nasal mucosa and submucosa was cut thoroughly in tissue culture medium consisting of RPMI 1640 (Sigma-Aldrich, Bornem, Belgium), containing 2 mM L-Glutamine (Invitrogen, Merelbeke, Belgium), antibiotics (50 IU/ml penicillin and 50 μg/ml streptomycin) (Invitrogen) and 0.1% BSA (Bovine Serum Albumin, Sigma). The tissue was passed through a mesh to achieve comparable fragments. The tissue fragments (+/- 0.9 mm^3^) were weighed and resuspended as 0.04 g tissue/1 ml tissue culture medium. The tissue was preincubated for 1 hour at 37°C, 5% CO_2 _with 1 μg/ml human myeloma IgE (Calbiochem, VWR International, Leuven, Belgium). After 3 washing steps the tissue fragments were resuspended in the appropriate amount of culture medium and 0.5 ml of this fragment suspension was dispensed per well of a 48 well plate. (BD Falcon, VWR, Leuven, Belgium). The fragment suspensions were stimulated with either culture medium (negative control), ε-chain specific anti-human IgE antibody (Dako Belgium N.V., Heverlee, Belgium), at 10 or 30 μg/ml (Dako Belgium N.V., Heverlee, Belgium), or 10 μM ionomycin (Calbiochem) for 30 minutes.

Supernatants were separated by centrifugation and stored immediately at -20°C until analysis of histamine, LTC_4_/D_4_/E_4 _and PGD_2_.

### Measurements of mediators in supernatants of stimulated tissue fragments

Concentrations of histamine, LTC_4_/D_4_/E_4 _and PGD_2 _were measured in tissue supernatants obtained after the stimulations using ELISA kits for Histamine (IBL Hamburg, Germany), LTC_4_/D_4_/E_4 _(Oxford Biomedical Research, Nuclilab BV, Ede, The Netherlands) and PGD_2 _(Cayman Chemicals, Ann Arbor, Michigan) following the instructions of the manufacture.

### Immunohistochemistry

Cryostat sections were prepared (6 μm) and mounted on SuperFrost Plus glass slides (Menzel Glaeser, Braunschweig, Germany), packed in aluminium paper and stored at -30°C until staining. Sections were immunohistochemically stained with the following antibodies: mouse anti human mast cell tryptase (clone G3, Chemicon International, Biognost, Heule, Belgium) and mouse anti human FcεRIα (clone CRA1, Gentaur, Brussels, Belgium). For immunohistochemical staining, specimens were fixed in Carnoy's Fluid (60% ethanol, 30% chloroform, 10% glacial acetic acid). Endogenous peroxidase activity was blocked with 0.3% hydrogen peroxide in TBS (Tris-buffered-Saline) containing 0.1% sodium azide for 20 minutes. The primary antibody or the negative control, consisting of the corresponding isotype control, was incubated for 1 hour and signal was detected using the LSAB+ technique conjugated with peroxidase according to the manufacturer's instructions (labelled streptavidin-biotin; Dako). The peroxidase activity was detected using AEC Substrate chromogen (Dako), which results in a red-stained precipitate. Finally the sections were counterstained with hematoxylin and mounted.

The number of positive cells was analysed using a magnification of 400× and scored by two independent observers who did not know the diagnosis and clinical data. The analyses included 10 relevant fields of the biopsy, and for each sample, the sum of positive cells/10 fields were scored.

### RNA preparation and real-time RT-PCR

Snap frozen tissue samples were placed in liquid nitrogen and thoroughly ground with a mortar and pestle and homogenized with Lysis Buffer (Bio-Rad Laboratories, CA, USA). Total RNA was purified using the Aurum™ Total RNA Mini Kit (Bio-Rad Laboratories, CA, USA) following manufacture's intructions. One microgram of total RNA was than reverse transcribed to generate cDNA with the iScript cDNA Synthesis Kit (Bio-Rad Laboratories, CA, USA) as instructed by the supplier. Expression of the IgERα-chain was determined using real-time PCR performed on an iCycler Real Time Detection System (Bio-Rad Laboratories, CA, USA). Primers and probes were purchased from Invitrogen (Merelbeke, Belgium) and contained the following sequences: IgERα (sense): 5'-TCTTCAGTGACTGGCTGCTCC-3', IgERα (antisense): 5'-GCTGGCCCTCCATCACC-3', IgERα-probe: FAM-5'-TCAGGCCTCTGCTGAG-3'-TAMRA [14]. PCR reaction contained 20 ng of cDNA, 300 nM of specific primers, 100 nM of TaqMan probe and 1× TaqMan Master mix (Bio-Rad Laboratories, CA, USA) in a final volume of 0.02 ml. Amplification program consisted in 1 cycle at 95°C for 10 min followed by 40 cycles at 60°C for 1 min and 95°C for 15 seconds. The expression of two housekeeping genes: Beta actin (ACTB) and Hydroxymethyl-bilane synthase (HMBS) was used to normalize for transcription and amplification variations among samples after a validation using the geNorm software as described previously [15]. The relative expression of the receptor was calculated with the qBase program (version 1.3.5, UGent, Belgium) based on the delta-C_T _relative quantification method. Results are shown as relative expression units per 20 ng cDNA (RNA based).

### Measurement of IgE in tissue homogenates

Snap frozen tissue specimens were weighed, and 1 ml of 0.9% NaCl solution was added per every 0.1 g tissue. The tissue was then homogenized with a mechanical homogenizer (B. Braun, Melsungen, Germany) at 1000 rpm for 5 min on ice as described previously [16]. After homogenization, the suspension was centrifuged at 3000 rpm for 10 min at 4°C and the supernatants separated and stored at -80°C until analysis. Immunoglobuline E was measured by the UNICAP system (Phadia, Uppsala, Sweden).

### Statistical analysis

Statistical analysis was performed using the Wilcoxon test (for paired comparisons). The Mann-Whitney U test was used for between-group (unpaired) comparisons. P values of less than .05 were considered as statistically significant. Correlations were made by using the Spearman rank correlation analysis.

## Results

### Mediator release after ex-vivo stimulations

A stimulation model was set up to stimulate inferior turbinate tissue (n = 8) and in larger quantities obtainable nasal polyp tissue (n = 8). IgE-primed nasal tissue fragments were stimulated with anti-IgE (10 μg/ml and 30 μg/ml) or ionomycin (10 μM) for 30 minutes. Stimulation resulted in a significant release and production of histamine, leukotrienes and PGD_2 _measured in the supernatants by ELISA. These mediators were released in a concentration-dependent manner, except for LTC_4_/D_4_/E_4 _in the inferior turbinate group (Table 2), where the difference between 10 and 30 μg/ml was not statistically significant.

**Table 2:** Overview of anti-IgE and ionomycin-induced release of histamine (ng/ml), LTC_4_/LTD_4_/LTE_4 _(ng/ml) and PGD_2 _(pg/ml) after 30 minutes in the nasal polyp (n = 8) and inferior turbinate group (n = 8).

	**Histamine (ng/ml**	**LTC_4_/LTD_4_/LTE_4 _(ng/ml)**	**PGD_2 _(pg/ml)**
*Nasal polyps*			

RPMI	24.1 (15.1–32.6)	0.0815 (0.048–0.11)	109 (66.5–221)

Versus	*P < 0.01*	*P < 0.01*	*P < 0.01*

Anti-igE 10 μg/ml	43.2 (28.1–55.5)	0.469 (0.348–0.816)	1960 (1518–4544)

Versus	*P < 0.01*	*P < 0.01*	*P < 0.01*

Anti-IgE 30 μg/ml	63.6 (44.8–75.5)	0.675 (0.561–1.21)	4949 (2991–6152)

Ionomycin 10 μM	130 (77.5–135)	3.40 (1.80–5.37)	2717 (1364–4298)

Versus baseline	*P < 0.01*	*P < 0.01*	*P < 0.01*

			

*Inferior turbinates*			

RPMI	8.5 (5.6–12.9)	0.036 (0.016–0.0395)	58.6 (40.2–88.2)

Versus	*P < 0.01*	*P < 0.01*	*P < 0.01*

Anti-igE 10 μg/ml	16.2 (12.0–20.2)	0.0655 (0.038–0.181)	840 (492–1269)

Versus	*P < 0.05*	*P = 0.44*	*P < 0.05*

Anti-IgE 30 μg/ml (n = 6)	28.1 (21.8–31.7)	0.0715 (0.058–0.331)	1669 (1311–1732)

Ionomycin 10 μM	27.6 (22.3–45.5)	0.361(0.24–0.525)	967 (548–1373)

Versus baseline	*P < 0.01*	*P < 0.01*	*P < 0.01*

Statistical analysis; Wilcoxon-test

Data are expressed as median +/- IQR.

**Figure 1:**
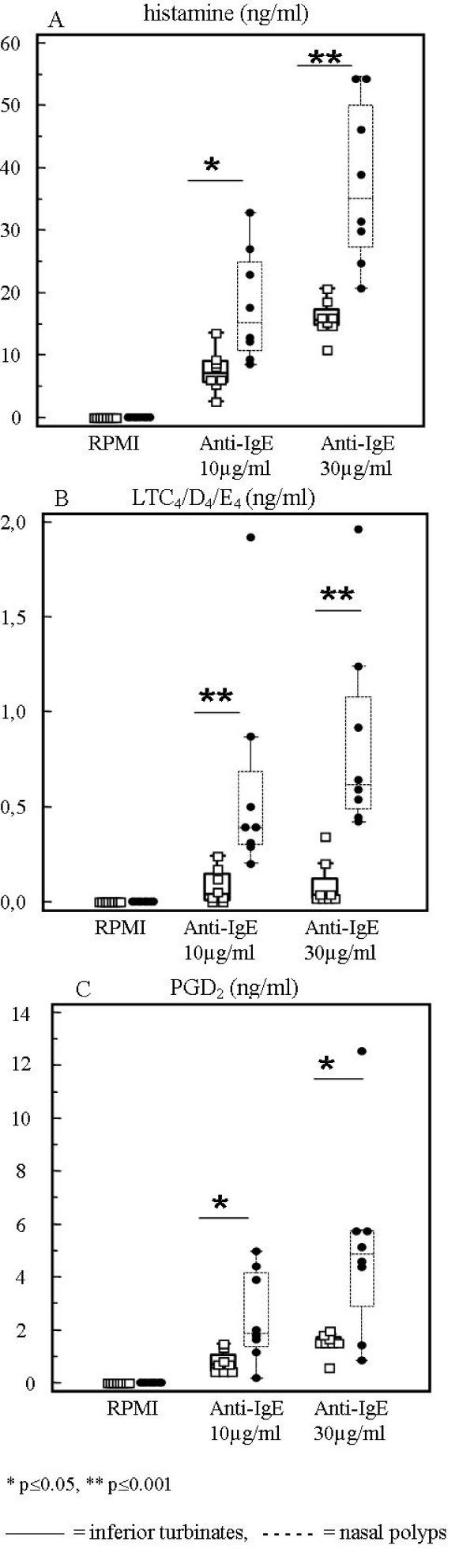
**Histamine (ng/ml) (A), LTC_4_/D_4_/E_4 _(ng/ml) (B) and PGD_2 _(ng/ml) (C) release after 30 minutes anti-IgE (10 μg/ml and 30 μg/ml) stimulation**. Comparison between nasal polyps (n = 8) and inferior turbinates (n = 8) after correction for baseline. The box-and-whisker plot represents the median, the lower to upper quartile, and the minimum to the maximum value, excluding outside and far out values, which are displayed as separate points. Statistical analyses were performed by using the Mann-Whitney U test. * p ≤ 0.05, ** p ≤ 0.001. [Black line] = inferior turbinates, [Dashed line] = nasal polyps.

After 30 minutes culture in medium alone, the spontaneous release of histamine and leukotrienes was significantly higher in nasal polyps compared to inferior turbinates (p < 0.01 and p = 0.03 respectively). However, the spontaneous release of PGD_2 _was not different between the two groups (p = 0.1). After correction for spontaneous release, the induced release of histamine, LTC_4_/D_4_/E_4 _and PGD_2 _was significantly higher in the nasal polyp group compared to the inferior turbinate group, both after stimulation with anti-IgE 10 μg/ml and anti-IgE 30 μg/ml (Fig 1).

### Immunohistochemistry

**Figure 2:**
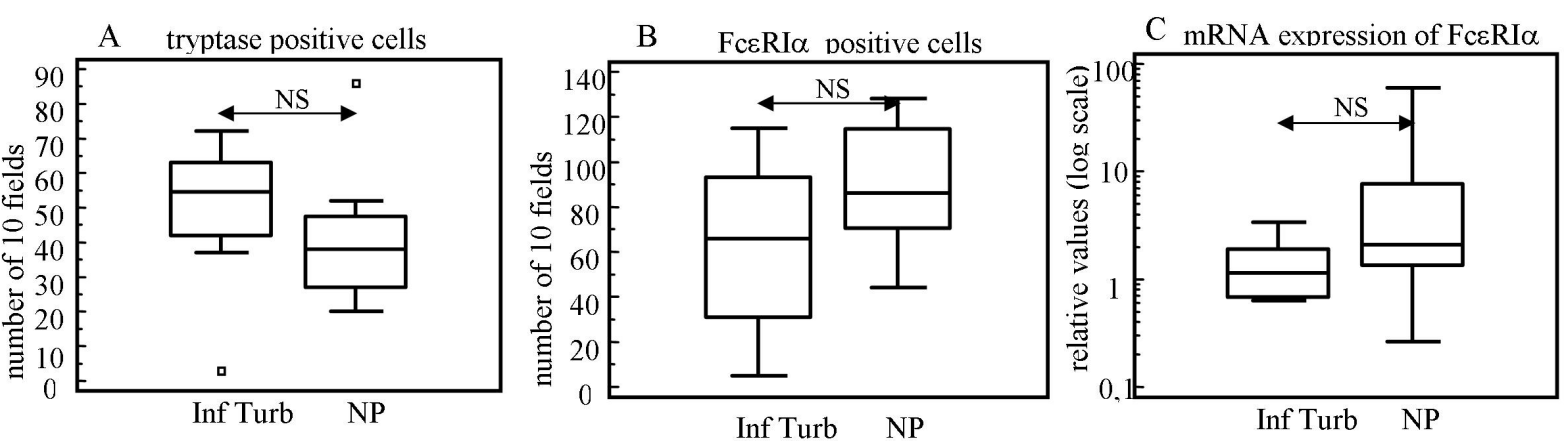
**Numbers of tryptase positive cells (A) and FcεRIα positive cells (B) in the inferior turbinate group (Inf Turb) (n = 8) and the nasal polyp group (NP) (n = 8), expressed as 10 scored fields (×400)**. The mRNA expression of FcεRIα in the inferior turbinate group and the nasal polyp group (C). The box-and-whisker plot represents the median, the lower to upper quartile, and the minimum to the maximum value, excluding outside and far out values, which are displayed as separate points. Statistical analyses were performed by using the Mann-Whitney U test. NS = Not Significant.

**Figure 3:**
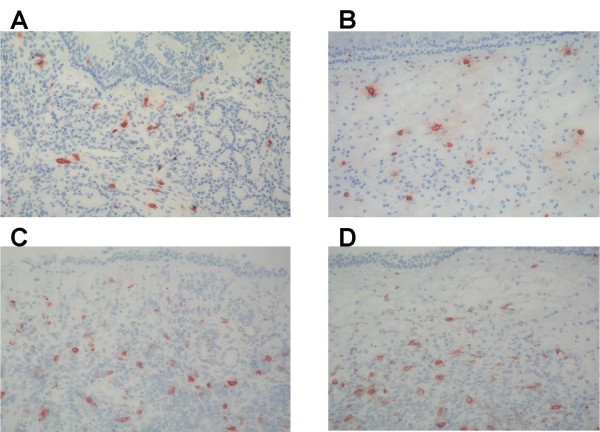
**Representative staining of tryptase positive cells in inferior turbinate tissue (A) and in nasal polyp tissue (B)**. Representative staining of FcεRIα positive cells in inferior turbinate tissue (C) and in nasal polyp tissue (D) (× 200).

In an attempt to explain the stronger response upon stimulation in nasal polyps versus inferior turbinates, mast cells and basophils were stained for tryptase and counted (Fig 2A), but no difference in the total numbers of mast cells in the nasal polyp group compared to the inferior turbinate group was detected. Furthermore, staining for FcεRIα showed no differences between the numbers of positive cells in both groups (Fig 2B). Representative stainings are shown in Fig 3.

### FcεRIα-chain mRNA

To study the expression of the high affinity IgE receptor, the amount of FcεRIα mRNA was quantified by RT-PCR in the nasal polyp and inferior turbinate groups. Equivalent FcεRIα mRNA levels were found in nasal polyps compared to inferior turbinates (Fig 2C).

### IgE in tissue homogenates

As it is described that the concentration of IgE is related[12] to the surface expression of FcεRI, and IgE concentrations are significantly higher in nasal polyps compared to controls [17], we studied the correlation between the IgE levels in tissue homogenates, and the release of histamine, LTC_4_/LTD_4_/LTE_4 _and PGD_2 _after anti-IgE challenge. Confirming earlier results, the concentrations of IgE were significantly higher in nasal polyps [97.6 (55.3–190.1) kUA/l] [median (IQR)] compared to inferior turbinates [10.3 (9.4–30.7) kUA/l] (p = 0.02). However, we were not able to demonstrate any correlation between the concentrations of IgE in nasal polyp homogenates and the amount of histamine release (r = 0.05, p = 0.9) (r = 0.1, p = 0.8), leukotriene release (r = 0.3, p = 0.4) (r = 0.4, p = 0.3) and PGD2 release (r = 0.3, p = 0.4) (r = 0.2, p = 0.5) after anti-IgE 10 μg/ml and anti-IgE 30 μg/ml stimulation respectively. Furthermore no correlation could be found between the concentrations of IgE in inferior turbinate homogenates and the amount of histamine release (r = 0.1, p = 0.7) (r = 0.5, p = 0.2), leukotriene release (r = 0.01, p = 1.0) (r = 0.5, p = 0.2) and PGD2 release (r = 0.02, p = 1.0) (r = 0.8, p = 0.1) after anti-IgE 10 μg/ml and anti-IgE 30 μg/ml stimulation respectively.

## Discussion

Until recently, cell systems used for exploration of mast cell biology have mainly been of rodent origin (the rat basophilic leukaemia cell line RBL-2H3, mouse bone marrow derived mast cells). The only human cell line available (HMC-1) [7] has been of limited usefulness due to the cells' stem cell factor independence, and inconsistent degranulation to IgE-dependent signals, presumably due to variable expression of the FcεRIα-subunit [7,18]. Other cell cultures, designated LAD 1 and 2, derived from bone marrow aspirates from a patient with mast cell sarcoma/leukemia, resemble CD34+-derived human mast cells with functional FcεRI and Fcγ RI receptors [8]. The use of bone marrow derived mast cells [19], umbilical cord blood derived mast cells [20], and foetal liver [21] or peripheral blood derived mast cells [19] have improved the models for studying mast cell biology [22]. Here, addition of certain interleukins such as IL-3, IL-6 or SCF to CD34+ progenitor cells made it possible to grow large numbers of committed mast cell precursors. However, mast cells display phenotypic heterogeneity depending on their tissue localisation, and any of those surrogate cell systems may prove not to represent the mast cells in a diseased tissue. It is therefore advantageous to study mast cells derived from human nasal mucosal, especially diseased tissue.

Pawankar et al [9] were able to isolate mast cells from inferior turbinates and study the IgE receptor, however, the number of mast cells remaining after stimulation is too little to perform meaningful mast cell activation and mediator release. Several studies [10,11] have made use of mast cells within digested nasal polyp tissue for stimulation, however these cells did not release histamine upon IgE receptor stimulation [23].

In this study we stimulated *ex-vivo *nasal tissue with anti-IgE to study mast cell activation and to compare the response in inferior turbinates and nasal polyps. By using whole tissue preparations, the cells remained in their natural environment, and unchanged surface receptor expression was maintained by omitting enzymatic digestion, thus closely mimicking the *in vivo *situation.

The stimulation with anti-IgE 10 μg/ml and anti-IgE 30 μg/ml resulted in a significantly higher production and release of mediators such as histamine, LTC_4_/D_4_/E_4 _and PGD_2 _compared to baseline, and these mediators were released in a concentration-dependent manner.

Although we measured mediators which are relatively restricted to mast cells such as histamine, PGD_2 _and LTC_4_/D_4_/E_4_, we could not totally exclude that other cells, which have been reported to express the IgE receptor, such as dendritic cells [24] and eosinophils [25], may also have been activated during this process. However, dendritic cells do not produce and release histamine, LTC_4_/D_4_/E_4 _or PGD_2, _and it is generally accepted that eosinophils are not a source of histamine and PGD_2_. Moreover, it has been shown that stimulation with human IgE and anti-IgE does not cause production of leukotriene C4 in eosinophils [26], demonstrating only mast cell activation in this setting.

Theoretically, basophils could contribute to the responses demonstrated here. It is, however difficult to discriminate between basophils and mast cells as effector cells. There are no reports about the number of basophils in nasal polyps in literature, suggesting a minor role of those cells in nasal polyps. Secondly, in the lamina propria of inferior turbinates of allergic patients, at baseline, the number of mast cells is at a median of 88%, with the percentages of basophils being as low as 3%. Only after allergen provocation, in the early phase, numbers of mast cells diminish sharply to a median percentage of 27% and basophils increase to 23% [27]. However, in the setting used here, mast cells most probably are the major contributors, as an influx of basophils in this *ex-vivo *model is impossible. Moreover, studies measuring mediators in nasal lavage fluid in an allergen-induced late-phase reaction revealed high levels of histamine but relatively low levels of products such as PGD_2_. Since histamine is released by mast cells and basophils, but prostaglandin D_2 _is not produced by basophils, these findings have implicated the basophils as an important contributor to histamine release in the late phase but not in the early phase[28,29]. In the here presented model, we thus most likely restrict the stimulation to mast cells.

Accessibility of nasal polyp tissue allows for easy assessment of interaction between different cell types in an inflammatory environment; however, the comparability of results obtained from nasal polyp stimulations to inferior turbinates was not studied so far. We therefore investigated the comparability of release of early mediators in nasal polyps versus inferior turbinates. We here demonstrate that the production and release of histamine, LTC_4_/D_4_/E_4 _and PGD_2 _was significantly and consistently higher in nasal polyps compared to inferior turbinates, both after stimulation with anti-IgE 10 μg/ml and anti-IgE 30 μg/ml.

The increased release of early phase mast cell mediators in nasal polyps could be due to the presence of a higher number of mast cells in nasal polyps. However, no difference in the total number of tryptase-positive cells in inferior turbinates compared to nasal polyps could be found by tryptase staining. Literature reports show contradictory findings; it is described that the number of epithelial mast cells in nasal polyps is elevated compared to controls[30,31] or that there is no difference in number of epithelial mast cells compared to controls[17,32]. In line with our findings, a recent study couldn't find any difference in the total number of mast cells between nasal polyps and inferior turbinates[33].

It is well described that mast cells in nasal polyps are mostly located in the stroma and are more degranulated compared to inferior turbinate mast cells [34,35]. Furthermore, stromal mast cells of dispersed nasal polyp tissue release higher amounts of histamine after anti-IgE stimulation compared to epithelial mast cells of the same tissue[36]. This underlines the heterogeneity of mast cells in different tissues and could point to a more activated status of polyp versus turbinate mast cells, and a higher sensitivity to external triggers. In line with our findings, levels of mast cell-derived mediators such as histamine and tryptase in nasal fluids from patients with nasal polyps are significantly higher than those observed in patients without nasal polyps [37]. Here we show that mast cells, even if partially degranulated in polyp tissue, still can produce and release higher amounts of mediators compared to the non-degranulated mast cells in inferior turbinates.

Having shown that the number of mast cells present was similar between polyp and turbinate tissue, we investigated whether the number of FcεRIα-positive cells was different between the two tissue types, but no difference was shown. The number of FcεRIα positive cells was higher than the number of tryptase positive cells, in both nasal polyps and inferior turbinates, which may be explained by the staining of other than mast cells, such as basophils, eosinophils [25] and dendritic cells [24].

Moreover, the FcεRIα chain expression at mRNA level did not demonstrate any difference in relative expression in nasal polyps compared to inferior turbinates. In the past, our group and others have described significantly higher levels of IgE in nasal polyp homogenates compared to controls [17,38]. As IgE levels may control cell surface levels of FcεRI [39], we expected higher levels of FcεRIα mRNA in the nasal polyps, which then could explain the increased release of mediators. However, in line with our results, other studies demonstrated that the presence or absence of IgE has no influence on the levels of mRNA for either alpha, beta, or gamma subunits of FcεRI [40,41].

In cord blood derived human mast cells, pre-incubation of mast cells for 4 days with IgE resulted in an enhancement of the IgE-binding ability of cells, and this was reflected by an increased surface expression of FcεRI. Moreover, this resulted in the elevated release of histamine, LTC_4 _and PGD_2 _in response to anti-IgE challenge[12]. However, we were not able to demonstrate a correlation between baseline IgE levels in nasal polyp and inferior turbinate homogenates and the amount of histamine, LTC_4_/LTD_4_/LTE_4 _or PGD_2 _release upon stimulation. Moreover, the release of mediators also was significantly different in polyp versus turbinate tissue after ionomycin stimulation, suggesting that the higher release in nasal polyps might be unrelated to the surface expression of FcεRI. Further studies need to clarify the mechanism behind this phenomenon.

## Conclusion

To conclude, a whole tissue nasal mucosal stimulation model was established which can be used to mimic the early phase of an allergic reaction both in nasal polyps and inferior turbinates.

We observed a significantly higher release of mast cell mediators after equivalent stimulation of nasal polyp tissues compared to inferior turbinates, the mechanism of which remains unclear. It is well recognized that mast cells with distinct functional and histochemical properties are present in human tissues [42,43]. The functional heterogeneity, the micro-environmental forces that dictate responsiveness and the impact of disease on mast cell response might be important in this process.

As high amounts of nasal polyp tissue are easier to access, and as nasal polyps and inferior turbinate tissue react in the same concentration- dependent manner to IgE- dependent triggers, nasal polyp tissue could be used to study the effect of inhibitors of the allergic early phase reaction in future settings.

## Abbreviations

LTC_4_/D_4_/E_4_: leukotrienes C_4_/D_4_/E_4_; PGD_2_: prostaglandin D_2_; IgE: Immunoglobulin E; FcεRIα: IgE receptor I; IL: interleukin; SCF: stem cell factor.

## Competing interests

This work was supported by a grant from the Flemish Scientific Research Board, FWO, Nr. A12/5-K/V-K17 to Claus Bachert, by a post-doctoral grant of the Research Foundation – Flanders (FWO) to Philippe Gevaert, and by an unrestricted research grant from GSK, Stevenage, United Kingdom

Furthermore, the authors declare that they have no competing interests.

## Authors' contributions

JP designed the stimulation model, included the patients, did the stimulation work and the statistics, and wrote the manuscript. GH designed the stimulation model, did the stimulation work, the ELISA's and the stainings. KA designed the stimulation model and helped to draft the manuscript and revised it critically. PG helped to draft the manuscript and revised it critically. CPN did the RT-PCR and helped to draft the manuscript. PVC helped to draft the manuscript and revised it critically. CB participated in the design and coordination of the study, helped to draft the manuscript and revised it critically. All authors read and approved the final manuscript.
